# Real-Time Assessment of *Staphylococcus aureus* Biofilm Disruption by Phage-Derived Proteins

**DOI:** 10.3389/fmicb.2017.01632

**Published:** 2017-08-24

**Authors:** Diana Gutiérrez, Lucía Fernández, Beatriz Martínez, Patricia Ruas-Madiedo, Pilar García, Ana Rodríguez

**Affiliations:** Instituto de Productos Lácteos de Asturias, Consejo Superior de Investigaciones Científicas Villaviciosa, Spain

**Keywords:** biofilm, *Staphylococcus aureus*, RTCA, MBEC_50_, LOABE, specific antibiofilm activity, phage lytic proteins, exopolysaccharide depolymerase

## Abstract

A current focus of research is the development of new tools for removing bacterial biofilms in industrial settings. Bacteriophage-encoded proteins, such as endolysins, virion-associated peptidoglycan hydrolases, and exopolysaccharide depolymerases, have been shown to be efficient against these structures. However, the current screening techniques for the identification of antibiofilm properties of phage-derived proteins have important shortcomings. The aim of this work was to use the rapid, reproducible and accurate technology “real-time cell analyzer” for screening and comparing the antibiofilm ability of four phage-derived compounds, three lytic proteins (LysH5, CHAP-SH3b, and HydH5-SH3b) and one exopolysaccharide depolymerase (Dpo7) against *Staphylococcus aureus* biofilms, which have been associated with recurrent contamination of food products. The data generated after biofilm treatment allowed for the calculation of different antibiofilm parameters: (1) the minimum biofilm eradicating concentration that removes 50% of the biofilm (ranging from 3.5 ± 1.1 to 6.6 ± 0.5 μM), (2) the lowest concentration needed to observe an antibiofilm effect (∼1.5 μM for all the proteins), and (3) the specific antibiofilm activity and the percentage of biofilm removal that revealed LysH5 as the best antibiofilm compound. Overall, this technology might be used to quickly assess and compare by standardized parameters the disaggregating activity of phage antibiofilm proteins.

## Introduction

In most environments, bacterial cells are commonly organized into mono or multi-species biofilms attached to a surface. These sessile communities generally have a specific tridimensional structure where the cells are entrapped in a matrix that can be composed of polysaccharides, proteins, teichoic acids, nucleic acids, and lipids (Flemming and Wingender, 2010). This particular lifestyle confers several advantages on the bacterial cells that ultimately enhance their tolerance to harsh environmental conditions. Thus, bacteria adhered to an inert surface or a tissue can avoid being washed away by water flow or the bloodstream and, perhaps more importantly, they are more resistant or tolerant to antibiotics, biocides and host defense mechanisms (Bridier et al., 2015; Olsen, 2015).

Bacterial biofilms have a huge impact on the environment, human health, and a wide variety of industrial processes (Hall-Stoodley and Stoodley, 2009; Van Houdt and Michiels, 2010; Martin et al., 2016). In fact, according to the National Institutes of Health, biofilms are responsible for more than 60% of microbial infections in humans and 80% of chronic infections (Bjarnsholt, 2013). Furthermore, these structures can cause severe economic losses in the food industry due to corrosion or obstruction of equipment, reduction in heat transfer and recurrent contamination of food products by spoilage or pathogenic bacteria, which represent an additional risk for public health (Myszka and Czaczyk, 2011). The resistance of biofilms to sanitation processes (Anand and Singh, 2013) together with a poor hygiene of food contact surfaces and equipment has repeatedly led to recurrent contamination of food products that can cause food-borne disease outbreaks. Particularly, the food-borne outbreaks related to the *Staphylococcus aureus* enterotoxins represented 6.4% of all the outbreaks in the EU in 2014 (EFSA and ECDC, 2016). Indeed, the ability to form biofilms enables this bacterium to colonize not only abiotic surfaces but also human tissues, making *S. aureus* one of the most important causative agents of nosocomial infections related to implanted medical devices (ECDC, 2013; Otto, 2013). The extracellular matrix of staphylococcal biofilms usually contains the exopolysaccharide poly-β-(1-6)-*N*-acetyl-glucosamine (PIA/PNAG), but biofilms lacking this component can be formed by the mediation of different surface proteins such as SasC, SasG, the clumping factor B (ClfB), the serine aspartate repeat protein (SdrC), the biofilm-associated protein (Bap), and the fibronectin/fibrinogen-binding proteins (FnBPA and FnBPB) (Speziale et al., 2014). Extracellular DNA (eDNA) is also an important component of the staphylococcal biofilm matrix and can participate in horizontal gene transfer (Montanaro et al., 2011).

Taking into account the serious human health risk posed by biofilms formed by pathogenic bacteria, it is imperative to design new antibiofilm strategies. A major goal is to develop novel antimicrobials that can circumvent biofilm resistance mechanisms, which include limited penetration of antimicrobial agents, reduced cell growth rate inside the biofilm and the presence of antimicrobial-tolerant persister cells, to name a few (Van Acker et al., 2014). Ideally, an antibiofilm agent should be able to penetrate the biofilm structure, degrade the matrix, and eliminate the bacteria (Rabin et al., 2015). This can be achieved by following two different approaches, which are inhibition of bacterial surface attachment and destabilization/disruption of mature biofilms (Miquel et al., 2016). Within this context, the use of bacteriophages represents a promising strategy to prevent and/or eliminate bacterial biofilms (Gutiérrez et al., 2016b). Bacteriophages are viruses that specifically target bacteria while being harmless to humans, animals and plants. The relentless rise in the antibiotic resistance of pathogenic bacteria has boosted a renewed interest in the utilization of phages as antimicrobials. Indeed, phages have been successfully used to fight against pathogenic bacteria in clinical, veterinary, food safety, and environmental contexts (O’Flaherty et al., 2009; García et al., 2010) and have shown promising results as antibiofilm agents (Gutiérrez et al., 2016b). Moreover, bacteriophages also constitute a source of novel antimicrobial enzymes that can be exploited to combat bacterial biofilms. For instance, phage-encoded lytic proteins, such as endolysins and virion-associated peptidoglycan hydrolases (VAPGHs), have shown great promise as antimicrobial agents (Rodríguez-Rubio et al., 2015). Furthermore, some authors have demonstrated that endolysins display antibiofilm activity (Sass and Bierbaum, 2007; Shen et al., 2013; Oliveira et al., 2014) and, in some cases, have proven their ability to kill persister cells (Gutiérrez et al., 2014). Additionally, phage-encoded proteins with polysaccharide depolymerase activity can be used for biofilm removal due to their ability to degrade the polysaccharide matrix (Gutiérrez et al., 2015; Pires et al., 2016).

Nowadays, the development of new antibiofilm agents can be hindered by the need for a reliable and accurate technology that allows the screening of the activity of these proteins. In a previous work, we demonstrated and validated that the xCelligence real-time cell analyzer (RTCA) equipment can be used to monitor biofilm formation and disruption in different bacterial species (Gutiérrez et al., 2016a). This was subsequently verified by Ferrer et al. (2017). This method offers several advantages compared to other techniques commonly used to test potential antibiofilm agents, like crystal violet staining or viable cell counting. Indeed, this system, which is based on impedance measurement recorded as cell index (CI), is non-invasive, label-free, fast, and reproducible. Here, we have used this technology to monitor biofilm elimination by different phage-derived proteins, in order to provide a rapid and standardized method to define and compare antibiofilm activities using different parameters such as minimum biofilm eradicating concentration that removes 50% of the biofilm (MBEC_50_), lowest observed antibiofilm effect (LOABE), specific antibiofilm activity, and percentage of biofilm removal.

## Materials and Methods

### Bacterial Strains, Culture Conditions, and Proteins

Four *S. aureus* strains (15981, ISP479r, IPLA1, and Sa9) were selected to test the antibiofilm activity of phage-derived proteins. *S. aureus* 15981 and ISP479r have a clinical origin (Valle et al., 2003), while *S. aureus* IPLA1 and Sa9 were isolated from food environments (García et al., 2007; Gutiérrez et al., 2012). As standard culture conditions, strains were grown in TSB (Tryptic Soy Broth, Scharlau, Barcelona, Spain) at 37°C with shaking, and frozen stocks (stored at -80°C) were plated onto TSB supplemented with 2% agar (TSA) and incubated for 24 h.

Phage-derived proteins LysH5 (García et al., 2010), CHAP-SH3b, HydH5-SH3b (Rodríguez-Rubio et al., 2012b), and Dpo7 (Gutiérrez et al., 2015) were purified as previously described. Prior to biofilm treatment assays, the buffer was exchanged to 50 mM sodium phosphate (NaPi) buffer (pH = 7.4) using the Kit “Zeba^TM^ Spin Desalting Columns, 7K MWCO, 5 mL” (Thermo Fisher Scientific, Madrid, Spain) following the supplier’s recommendations; afterward, the supernatants were filtered using 0.22 μm PES membrane filters (VWR, Spain). The amount of protein was quantified by using the Quick Start^TM^ Bradford Protein Assay kit (Bio-Rad, Madrid, Spain). Quantification of the lytic activity of proteins LysH5, CHAP-SH3b, and HydH5-SH3b was performed by the turbidity reduction assay against planktonic *S. aureus* Sa9 cells (Obeso et al., 2008) and by MIC (minimal inhibitory concentration) determination assay which was carried out by the conventional broth microdilution technique in TSB (CLSI, 2015). The specific lytic activity of the lytic proteins was calculated as the decrease in the absorbance (OD_600_ nm) per μM of protein and per minute (ΔOD × μM^-1^ × min^-1^); the MIC was set at the concentration (μM) where no bacterial growth was observed and calculated as the mode of three independent biological replicates.

### Monitoring of Biofilm Formation

Biofilm formation was carried out as described previously (Gutiérrez et al., 2016a). Briefly, standardized grown cultures were diluted down to 10^7^ CFU/ml in fresh TSBG broth (TSB supplemented with 0.25% glucose). Then, 100 μl of this suspension were poured into 16-well E-plates (∼10^6^ cells/well), which were then connected to the xCelligence RTCA-DP (ACEA Biosciences Inc., San Diego, CA, United States) holder pre-warmed at 37°C. Biofilm formation was monitored by recording impedance measurements of the CI every 10 min for 8 h. Biofilm formation was performed in three independent biological replicates.

### Monitoring the Antibiofilm Effect of Phage-Derived Proteins

The effect of phage-encoded proteins over 8 h-preformed biofilms was determined after addition of 100 μl of increasing concentrations of the four phage-derived proteins (0.04–12 μM) diluted in TSBG. Monitoring of the CI variations at 37°C was recorded every 10 min for an extra period of 16 h. A sample with NaPi buffer 50 mM pH = 7.4 diluted in TSBG was also included as a control. The RTCA software 1.2.1 (ACEA Biosciences Inc.) was used for further analysis of the data obtained. First, a time-point normalization of the CI was performed 10 min after starting the treatment (at this time point, the value of the “normalized CI” is 1). These normalized CI values were used to calculate the percentage of biofilm removal referred to the control values after 16 h of treatment. In a next step, all data were referred to the control value (“baseline CI”) by subtracting the normalized CI of each sample from the normalized CI of the control; thus, the value of the “baseline normalized CI” for the control is always 0. From these data, the specific antibiofilm activity was determined as the decrease in the baseline normalized CI per mM of protein and per minute (Δbaseline normalized CI × mM^-1^ × min^-1^) in the linear range of the curve. Moreover, these baseline normalized CI values were also used to calculate a dose–response curve (DRC) of the baseline normalized CI at a particular time *vs* concentration (% of baseline normalized CI × time *vs* μM of protein). The time was set according to the following two criteria: first, there should be a clear difference in the effect of the concentrations under study, and, second, the DRC should fit *R*^2^ > 0.98 at the selected time point. The resultant sigmoidal DRC was calculated at this time for the three biological replicates independently; the MBEC_50_ was finally determined by the RTCA software and expressed as the mean ± standard deviation. The MBEC_50_ was defined previously as the concentration causing 50% reduction in the biofilm metabolic activity (Budzynska et al., 2011); herein, MBEC_50_ represents the minimum biofilm eradicating concentration of protein that decreases the normalized CI × time by 50%. Moreover, the LOABE value was defined as the lowest concentration of protein tested that produced a detectable antibiofilm effect. This value is equivalent to the LOAEL (lowest observed adverse effect level) calculated in epidemiological or toxicological animal studies (Jeffery et al., 2004; Valdés et al., 2015).

Additionally, traditional staining with crystal violet was carried out to determine the total biomass adhered to the gold microelectrodes of the E-plate wells after protein treatment (16 h) with the modifications described previously (Gutiérrez et al., 2016a). The percentage of biofilm removal was calculated in reference to the control wells.

### Statistical Analysis

The SPSS Statistics for Windows V. 22.0 (IBM Corp.) package was used to perform two assessments: (1) within each strain, antibiofilm differences among phage-derived proteins, and (2) within each phage-derived protein, antibiofilm differences among strains. The data were expressed as the mean ± standard deviation and the differences were determined by one-way analysis of variance (ANOVA) followed by the Student–Newman–Keuls test for comparison of means at a level of significance *p* < 0.05.

In addition, the LOABE value was calculated for each protein using the values obtained from DRC at the selected time point by comparing (two by two, by one-way ANOVA) the baseline normalized CI values obtained for consecutive protein concentrations; the LOABE was defined as the first point that showed statistical differences (*p* < 0.05).

On the other hand, linear regression equations among different numeric parameters (percentage of biofilm reduction calculated using normalized CI *vs* absorbance 495 nm, and absorbance 495 nm *vs* specific antibiofilm activity) were calculated in order to obtain the coefficients of determination (*R*^2^) which show how well data fit to the linear regression equations.

## Results

### Real-Time Monitoring of Biofilm Disruption by Phage-Derived Proteins

Based on our previous experience, strain *S. aureus* 15981 was chosen for the screening of the effective concentration range of four phage-derived proteins as antibiofilm agents: the three lytic proteins LysH5, CHAP-SH3b and HydH5-SH3b and the polysaccharide depolymerase Dpo7. Indeed, this strain is a strong biofilm former and had previously shown a good correlation between RTCA data and other methods (crystal violet staining and viable cell counting; Gutiérrez et al., 2016a).

To establish the concentration range for the four proteins under study, we monitored *S. aureus* 15981 biofilm disruption with the xCelligence RTCA system. To do that, biofilms were grown in the E-plates for 8 h until early stationary phase was reached. At this point, increasing concentrations (from 0.04 to 12 μM) of the four purified proteins were added to the wells. Immediately after adding the proteins, there is a noticeable increase in the impedance signal which seems to be proportional to the protein concentration. However, this increase was actually due to the buffer used for protein storage (NaPi, 50 mM pH = 7.4), and not to protein activity. Indeed, treatment of 8 h old preformed biofilms with different dilutions of the buffer alone in TSB led to an initial increase in the impedance signal, followed by a stabilization of the values after 10 min (data not shown). As a result, to avoid the effect of the buffer and ensure that all the values obtained are protein dependent, normalization of the impedance signal was performed after 10 min. Thus, at this time (10 min) data were transformed to “Baseline normalized CI” and measurements after this point, were used to calculate the activity of the different proteins (Supplementary Figure S1).

**FIGURE 1:**
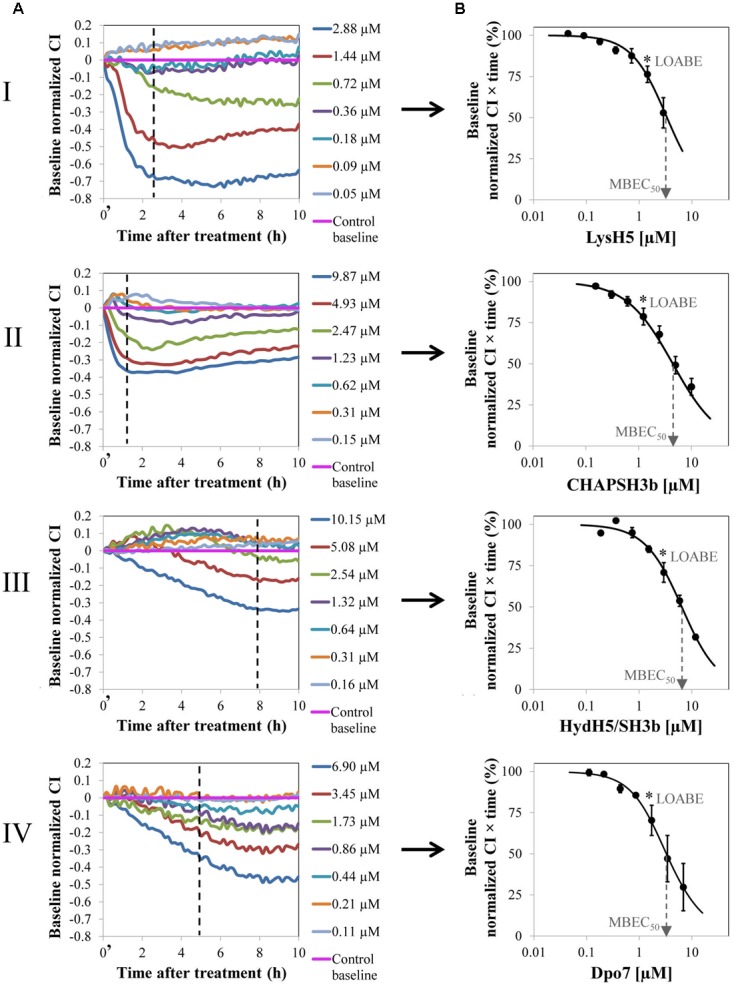
Removal of 8 h-old preformed biofilm of *S. aureus* 15981 treated with increasing concentrations of phage-derived proteins: LysH5 (I), CHAP-SH3b (II), HydH5/SH3b (III), and Dpo7 (IV). **(A)** Variation of the baseline normalized cell index (CI) during biofilm treatment for one representative biological replicate out of three showing the same trend. Time *t* = 0′ in the *x*-axis represents the time 10 min after the beginning of the treatment. **(B)** Dose–response curve (DRC) fitted into a sigmoidal curve (*R*^2^ > 0.98) obtained by representing the baseline normalized CI *vs* protein concentration at a particular time (dashed line in **A**); each point in the curve corresponds with the mean (±standard deviation) calculated from three biological replicates. The dashed arrow indicates the MBEC_50_ value and the asterisk the LOABE value (see **Table 1**).

The treatment of 8-h biofilms of *S. aureus* 15981 with increasing concentrations of the phage-derived proteins resulted in a dose-dependent reduction of the baseline normalized CI due to biofilm removal (**Figure 1A**). The minimum value was reached after 2 h for LysH5 and CHAP-SH3b and 7–8 h for HysdH5/SH3b and Dpo7; these lowest baseline normalized CI values, indicating the lowest remnant biofilm biomass, correspond with the highest protein concentration. After this time point, all impedance values remained unaltered.

### Calculation of the MBEC_50_

To compare the efficacy of the four proteins, a numerical parameter from the baseline normalized CI *vs* time curves was established after defining the best time point for each protein (dashed line, **Figure 1A**). Then, at this time, the baseline normalized CI values were represented as a function of the protein concentration resulting in a DRC (**Figure 1B**). The best time point to obtain the DRC was set according to two criteria: first, the effect shown by the protein concentrations should be clearly differentiated, and, second, the resulting DRC should fit *R*^2^ > 0.98. The obtained results confirmed that an increase in protein concentration led to a gradual reduction in the normalized CI values and that this decrease was higher at higher concentrations of the compounds tested. Therefore, the DRCs clearly demonstrate a dose-dependent antibiofilm effect of the proteins tested against 8 h-biofilms formed by *S. aureus* 15981. These curves subsequently allowed the calculation of the MBEC_50_ and the LOABE values corresponding to the four proteins (**Figure 1B** and **Table 1**). MBEC_50_ values ranged from 3.5 ± 1.1 to 6.6 ± 0.5 μM, obtained respectively for LysH5 and HydH5-SH3b, and showed statistically significant differences among the four proteins tested (*p* < 0.05). Interestingly, the biofilm removal activity of the exopolysaccharide depolymerase Dpo7 and CHAP-SH3b was similar to that observed for LysH5 (*p* ≥ 0.05). Moreover, within the studied concentrations range, the LOABE values were similar for all four proteins (∼1.5 μM) (**Figure 1B** and **Table 1**). This result seemed to indicate that concentrations below the LOABE values did not alter the biofilm structure, neither lysing the cells in the case of the lytic proteins, nor removing the polysaccharide matrix in the case of Dpo7.

**Table 1:** Activity of phage-derived proteins.

	Antibiofilm effect		Lytic activity	
	MBEC_50_ (μM)	LOABE (μM)	Specific activity (ΔOD × μM^-1^ × min^-1^)	MIC (μM)
LysH5	3.5 ± 1.1^a^	1.4	2.5 ± 0.5^a^	2.6
CHAP-SH3b	4.4 ± 1.2^a^	1.2	1.7 ± 0.6^a^	3.1
HydH5/SH3b	6.6 ± 0.5^b^	1.5	0.6 ± 0.2^b^	5.4
Dpo7	3.7 ± 1.5^a^	1.7	–	–

*MBEC_50_ (μM) and LOABE (μM) of the different proteins against 8 h-old preformed biofilms of *S. aureus* 15981. The lytic activity of the proteins was calculated against planktonic cells and is expressed as specific lytic activity (ΔOD × μM^-*1*^ × min^-*1*^) and MIC (μM). MBEC_50_ and specific lytic activity values represent mean ± standard deviation of three biological replicates. MIC is expressed as the mode of three independent biological repeats. Values in the same column having distinct letter are statistically different (*p* < 0.05) according to the ANOVA and SLK *post hoc* comparison test*.

To study a potential relationship between antimicrobial and antibiofilm activities for the phage lytic proteins (LysH5, CHAP-SH3b, and HydH5-SH3b), we determined the specific lytic activity of each protein against *S. aureus* 15981. The results of this assay revealed that LysH5 and CHAP-SH3b showed the highest specific lytic activity (2.5 and 1.7 units, respectively), while HydH5-SH3b exhibited a lower specific activity (0.6 units) (**Table 1**). This result is in good agreement with the lower antibiofilm activity observed by RTCA for this protein in comparison with the other two. Moreover, MIC values were further calculated and were surprisingly similar to the MBEC_50_ values obtained for each protein (**Table 1**).

### Spectrum of Activity of Antibiofilm Proteins

**FIGURE 2:**
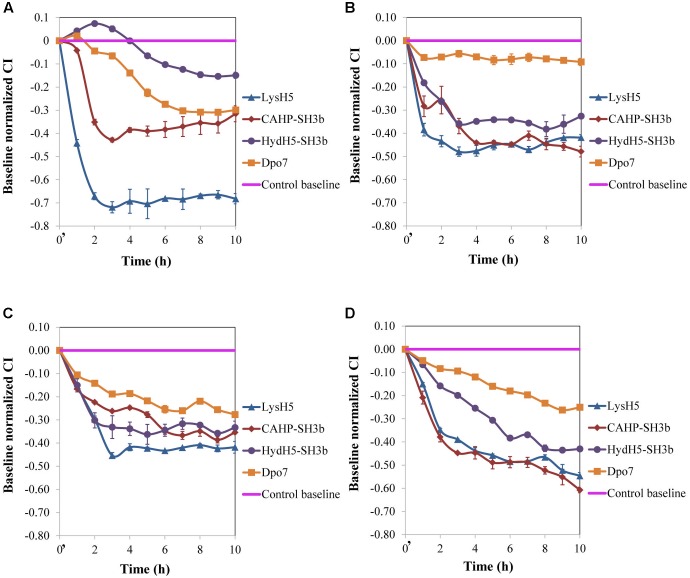
Removal of 8 h-old preformed biofilms of *S. aureus* **(A)** 15981, **(B)** ISP479r, **(C)** IPLA1, and **(D)** Sa9, treated with ∼7 μM of proteins LysH5, CHAP-SH3b, HydH5-SH3b, and Dpo7. Mean ± standard deviation was calculated for each value of normalized CI throughout incubation from three biological replicates. Time *t* = 0′ in the *x*-axis represents the time 10 min after the beginning of the treatment.

Our results, so far, confirmed the potential of the xCelligence RTCA system for screening antibiofilm compounds. In a step forward, this procedure was used to assess the spectrum of activity of the selected antibiofilm agents for other *S. aureus* isolates. Three additional staphylococcal strains with different ability to form biofilms were used: ISP479r is a strong biofilm producer and IPLA1 and Sa9 are weak biofilm producers. The ability of these strains to produce stronger (15981 and ISP479r) or weaker (Sa9 and IPLA1) biofilms was established after measuring the CI for 8 h. In general, the maximum CI values for strong biofilm producers in stationary phase were over 2 whereas the values obtained for weak biofilm producers were around 1 (data not shown). Preformed biofilms (8 h-old) of *S. aureus* 15981, ISP479r, IPLA1, and Sa9 were treated with each protein for 16 h at 37°C using a protein concentration (∼7 μM) which was twice the MBEC_50_ for LysH5, CHAP-SH3b and Dpo7; in the case of HydH5-SH3b, the MBEC_50_ value (6.6 μM) was used due to the impossibility to purify a more concentrated stock of this last protein. The four assayed staphylococcal strains turned out to be sensitive to all proteins since a decrease in the baseline normalized CI can be observed in all cases (**Figure 2**). The baseline normalized CI data were then used to define the antibiofilm specific activity (Δbaseline normalized CI × mM^-1^ × min^-1^), which resulted in values ranging from 0.08 ± 0.01 to 0.79 ± 0.02 units (**Table 2**). These values reflect the sensitivity of a given strain to each protein along time, allowing comparison of the activity spectrum among different proteins. Moreover, taking together the data obtained with different strains, we could establish similarities and differences in the activity spectra of the four proteins. When the data obtained for the four strains were combined, differences among the treatment with the four proteins (*p* < 0.05) were easily denoted. For instance, LysH5 showed the highest specific antibiofilm activity against all strains, while the lytic proteins derived from the VAPGH HydH5 possessed a medium specific antibiofilm activity. In contrast, the lowest specific antibiofilm activity against all strains was observed for the exopolysaccharide depolymerase Dpo7 (Supplementary Figure S2). Interestingly, the activity of the proteins against the biofilms was not dependent on the robustness of the biofilm since similar values of activity were obtained against strong and weak biofilms for each protein (**Table 2**).

**Table 2:** Specific antibiofilm activity (Δbaseline normalized CI × mM^-1^ × min^-1^) of the different proteins against 8 h-old biofilms formed by four *S. aureus* strains (15981, ISP479r, IPLA1, and Sa9).

Protein (μM)	*S. aureus* strains			
	15981	ISP479r	IPLA1	Sa9
LysH5 (7 μM)	0.79 ± 0.03^a,A^	0.47 ± 0.04^b,A^	0.35 ± 0.02^c,A^	0.41 ± 0.02^b,A^
CHAP-SH3b (7 μM)	0.38 ± 0.03^a,B^	0.21 ± 0.03^b,B^	0.25 ± 0.02^c,B^	0.45 ± 0.01^d,B^
HydH5/SH3b (7 μM)	0.09 ± 0.04^a,C^	0.28 ± 0.01^b,B^	0.36 ± 0.01^c,A^	0.18 ± 0.01^d,C^
Dpo7 (7 μM)	0.11 ± 0.01^a,C^	0.08 ± 0.01^b,C^	0.15 ± 0.01^c,C^	0.10 ± 0.01^d,D^

*Values represent mean ± standard deviation of three biological replicates. Values having distinct lower case letter in the same row indicate that the protein treatments are statistically different (*p* < 0.05) among the strains, while values with distinct capital letter in the same column indicate statistical differences (*p* < 0.05) between protein treatments within each strain, according to the ANOVA and SLK *post hoc* comparison test*.

**FIGURE 3:**
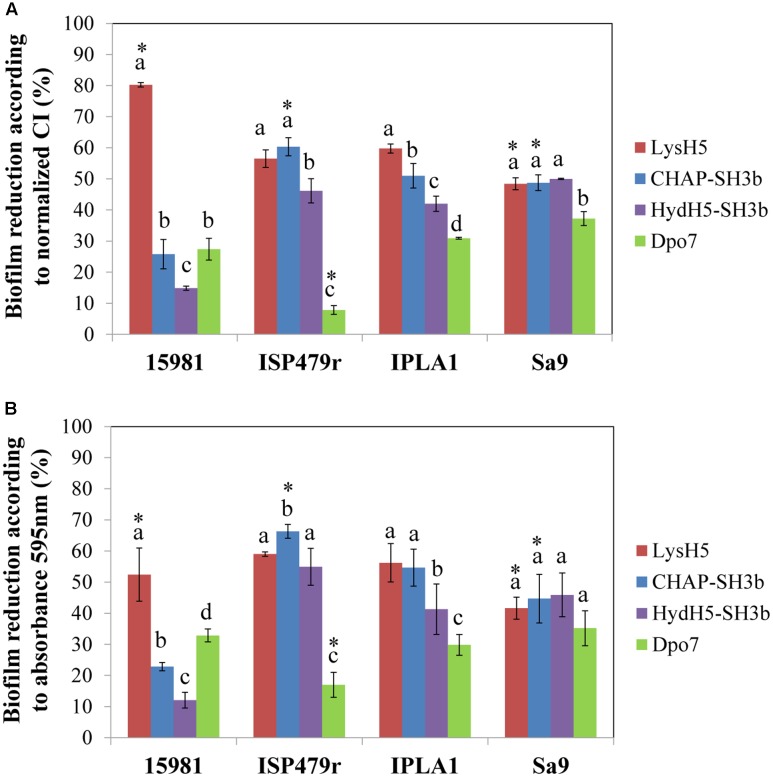
Comparison of the RTCA-based technique and conventional crystal violet staining expressed as biofilm reduction (%) after 16 h of treatment at 37°C. Data were calculated for each biofilm-producing staphylococcal strain treated with the protein under study using the values obtained by measuring **(A)** the normalized CI or **(B)** crystal violet staining (absorbance at 595 nm). Bars represent mean ± standard deviation of three biological replicates. Bars within each strain having distinct lower case letter indicate that the protein treatment is statistically different (*p* < 0.05); for the each pair strain/protein, an asterisk represents statistical differences between the biofilm reduction (%) obtained using the normalized CI and the absorbance 595 nm values (*p* < 0.05; ANOVA and SLK *post hoc* comparison).

Finally, to confirm a correlation between the data obtained with the RTCA system and the standard crystal violet staining procedure, the percentage of biofilm reduction was calculated at the point corresponding to 16 h of treatment. On the one hand, the calculation was performed using the normalized CI values before adjusting the parameters to the control baseline (**Figure 3A**) and, on the other hand, the total biomass was determined by crystal violet staining measuring the absorbance at 595 nm (**Figure 3B**). When comparing both results, in general, there were no statistically significant differences between them (*p* ≥ 0.05) indicating that the RTCA system can be used to calculate the percentage of biofilm reduction, similarly to the standard crystal violet method currently used. Indeed, both methods are measuring the amount of remnant biomass after treatment with phage-related proteins at a given point. In fact, there was a good correlation between the data obtained with the two systems (Supplementary Figure S3). However, as expected, there was not a good correlation between the percentages of biofilm reduction calculated with crystal violet staining and the specific antibiofilm activity (data not shown). This is not surprising since the latter is representative of death kinetics where three parameters are taken into account: the decrease in impedance, the concentration of protein used in the experiment and the time spent to reach the lowest impedance value.

## Discussion

Over the last few years, the study of phage lytic proteins for the treatment of infectious diseases has attracted great interest (Fischetti, 2010). More recently, they have even been proposed as disinfectants for the food industry (Gutiérrez et al., 2016b). Indeed, there is evidence that many of these proteins are effective in animal models of infection due to the fact that they also have antimicrobial activity against biofilms formed by these bacteria (Singh et al., 2010; Díez-Martínez et al., 2013; Schmelcher et al., 2015).

A major shortcoming in the study of biofilms is the lack of consensus among the diversity of techniques used to grow and study these structures. When working in static conditions, biofilm formation in microtiter plates is certainly the most common method used, to test both biofilm formation and the antibiofilm activity of different compounds. However, this technique involves endpoint quantification, and although amount of biofilm biomass, cell viability or metabolic activity can be determined by easy and cheap staining techniques, the results obtained sometimes are not reproducible and are person or laboratory dependent (Azeredo et al., 2017). Real-time monitoring would, however, overcome this issue as it allows quantification of changes throughout biofilm after treatment, being highly reproducible between laboratories (Gutiérrez et al., 2016a; Ferrer et al., 2017). The goal of this study was to design a method to determine the antibiofilm activity of phage-derived proteins against staphylococcal biofilms. This measurement should be useful for the assessment of both phage lytic proteins and other phage-derived proteins such as exopolysaccharide depolymerases. In addition, a definition of activity units was proposed to easily compare among different proteins. We had previously characterized several phage lytic proteins (CHAP-SH3b and HydH5-SH3b) with enhanced staphylolytic activity, derived from the VAPGH HydH5 and lysostaphin (Rodríguez-Rubio et al., 2012b). In addition, we determined that endolysin LysH5, encoded by the *S. aureus* phage vB_SauS-phiIPLA88, reduced staphylococcal sessile counts by 1–3 log units in polystyrene adhered biofilms (Gutiérrez et al., 2014). Similarly, the exopolysaccharide depolymerase Dpo7, encoded by the *Staphylococcus epidermidis* bacteriophage vB_SepiS-phiIPLA7 was able to remove up to 90% of biomass in staphylococcal biofilms formed by polysaccharide-producing strains (Gutiérrez et al., 2015). To date, the peptidoglycan hydrolase activity of phage lytic proteins is determined spectrophotometrically (turbidity reduction assay) and the definition of activity units is based on the decrease of optical density. Thus, one enzymatic unit would represent the amount of protein able to reduce the OD_600_ of a bacterial suspension by 50% in 15 min (Briers et al., 2007). Moreover, the reduction in optical density over time (minutes or hours) can be used to calculate the hydrolysis rate known as “specific lytic activity” that is reported as ΔOD × time^-1^ × mg^-1^ of lytic protein. In addition to the turbidity reduction method, there are other assays such as time-kill curve (viable counts), zymogram, spot-on-lawn, MIC and minimum bactericidal concentration, that can be performed to test the lytic activity of phage proteins (Nelson et al., 2012). For measuring exopolysaccharide depolymerase activity against bacterial biofilms there is no standardized approach beyond the reduction in total biomass of biofilms quantified by crystal violet staining (Gutiérrez et al., 2015). A different approach is the measurement of β-hexosaminidase activity of the bacterial exopolysaccharide depolymerase DspB against a synthetic substrate (Kaplan et al., 2003).

Here, we used the RTCA methodology to infer the decrease in impedance as a direct measurement of antibiofilm activity. Previously, the impedance-based system proved to be an accurate technology to measure the ability of staphylococcal strains to form biofilm, showing a high correlation with values obtained by standard approaches such as crystal violet staining and bacterial cell counts, as well as with those obtained upon other abiotic surfaces (polystyrene and stainless steel; Gutiérrez et al., 2016a). The RTCA method was also validated for biofilm removal using bacteriophages and phage-derived proteins (Gutiérrez et al., 2016a) and some antibiotics (Ferrer et al., 2017).

In the current work, we have shown that this methodology is useful to perform a reliable and quick screening of proteins with antibiofilm activity and also to test the sensitivity of different staphylococcal strains to these proteins. Our results showed MBEC_50_ values in the micromolar scale (3.5 μM) for LysH5, CHAP-SH3b, and Dpo7. As expected, the highest MBEC_50_ values against *S. aureus* 15981 were obtained for HydH5-SH3b that showed the lowest antimicrobial activity against planktonic cells. This chimeric protein also had lower lytic activity against planktonic cells of another *S. aureus* Sa9 strain (Rodríguez-Rubio et al., 2012a). However, this correlation does not necessarily imply that antibiofilm activity is equivalent to lytic activity. For instance, diffusion of the protein into the biofilm, which is determined by size and charge, would limit its activity (Zhang et al., 2011). This limited activity due to diffusion of the protein, is also highlighted due to the MBEC_50_ values obtained for the lytic proteins were equal to onefold the MIC, indicating that a higher concentration of the protein is needed to remove biofilms. Similar results were obtained using a novel antimicrobial compound (mul-1867) against *Pseudomonas aeruginosa* biofilms, where a concentration of onefold the MIC was able to remove the 50% of the biofilm (Tetz et al., 2016).

## Conclusion

In conclusion, the method proposed here will allow the easy screening, in static conditions, of specific settings for biofilm removal (pH, temperature, ionic strength). Moreover, a high number of strains can be simultaneously tested for their sensitivity to the proteins since there are several RTCA systems available on the market that allow testing up to 1536 samples in the same experiment. Taking into account the small size of the equipment, the RTCA device can easily fit into any incubator to perform experiments at different temperatures (ranging from 15 to 40°C). It is worth noting that, to make the most of this technology, it would be helpful to develop the RTCA xCelligence systems to allow for testing of biofilm formation and removal under dynamic conditions. Unfortunately, to date this technology only allows testing under static conditions. Of note, the real-time recording of impedance values allows determining the precise time where the maximum elimination of the biofilm is achieved, and it is also suitable for the calculation of parameters as important as the MBEC_50_ and LOABE values, which represent the protein concentration needed to eliminate the 50% of the biofilm and the minimum concentration to produce an alteration in the biofilm, respectively. This technique also permits determining the specific antibiofilm activity, which is an indicator of biofilm degradation dependent on the incubation time and on the protein concentration. The results obtained in this study show that the percentage of biofilm removal values estimated with RTCA correlate with those obtained using a standard staining method, while providing additional information. Taken together, the values of the effective dose for biofilm removal and the spectrum of activity against a strain collection facilitate the evaluation of antibiofilm properties of these proteins. This would also be an advantage to speed up the process by selecting optimal combinations of phage lytic proteins, exopolysaccharide depolymerases or other antimicrobial compounds against biofilms. In this sense, the study of such synergistic effects should be a focus of further research.

## Author Contributions

DG, LF, BM, PR-M, PG, and AR conceived and designed the experiments. DG performed the experiments. DG, PR-M, PG, and AR analyzed the data. DG, LF, BM, PR-M, PG, and AR wrote the paper.

## Conflict of Interest Statement

The authors declare that the research was conducted in the absence of any commercial or financial relationships that could be construed as a potential conflict of interest.
