# Supplementary material for: Real-Time Assessment of Staphylococcus aureus Biofilm Disruption by Phage-Derived Proteins
Source: Front Microbiol. 2017 Aug 24;8:1632. doi: 10.3389/fmicb.2017.01632 (PMC5573737; doi:10.3389/fmicb.2017.01632)
Supplement: Supplementary file 1 [file Data_Sheet_1.PDF]

Supplementary Material

Real-time assessment of *Staphylococcus aureus* biofilm disruption by phage-derived proteins

Diana Gutiérrez*, Lucía Fernández, Beatriz Martínez, Patricia Ruas-Madiedo, Pilar García and Ana Rodríguez

*** Correspondence:** Dr Diana Gutiérrez, dianagufer@ipla.csic.es

1 SUPPLEMENTARY FIGURES

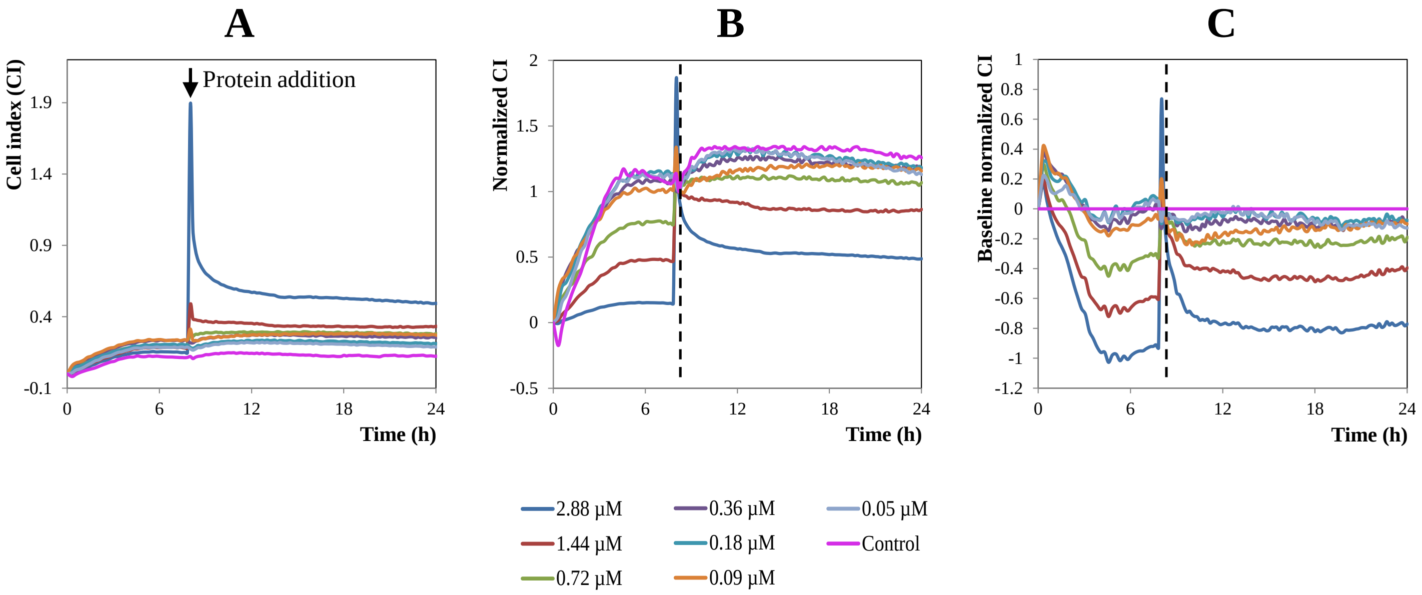

Figure S1. Example of an antibiofilm assay using the real-time impedance system and further software processing. (A) Generic real-time impedance trace (measured as CI) obtained after the treatment (for 16 h) of 8 h-old biofilms of S. aureus 15981 with different concentrations of LysH5. (B) Normalization of the data 10 min after exposure to the protein (normalized CI); at this point, the values of all treatments were relativized to 1. This data were used to calculate the percentage of biofilm removal compared to the control. (C) Then, all data were referred to the control value by subtracting the normalized-CI of each sample from the normalized-CI of the control (= baseline normalized cell index); Thus, the value of the “baseline normalized CI” for the control is always 0. These final data were used to obtain the specific antibiofilm activity and represent the DRC which led to the calculation of MBEC50 and LOABE values.

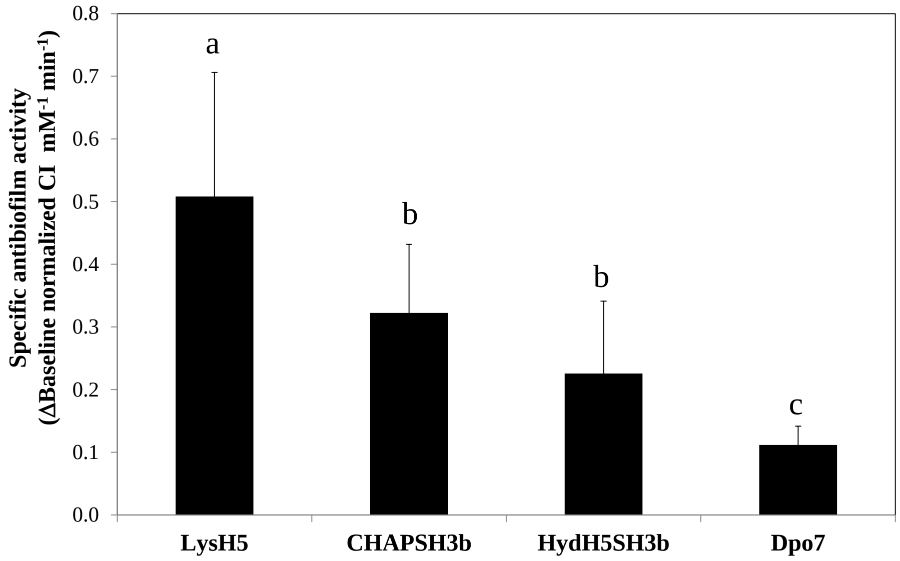

Figure S2. Statistical analysis of the specific antibiofilm activity. Activity of the phage-derived proteins combining the data obtained for the four strains against staphylococcal biofilms. Values represent mean ± standard deviation of twelve biological replicates. Values having distinct lower case-letter indicate statistically differences ($p<0.05$) according to the ANOVA and SLK post-hoc comparison test.

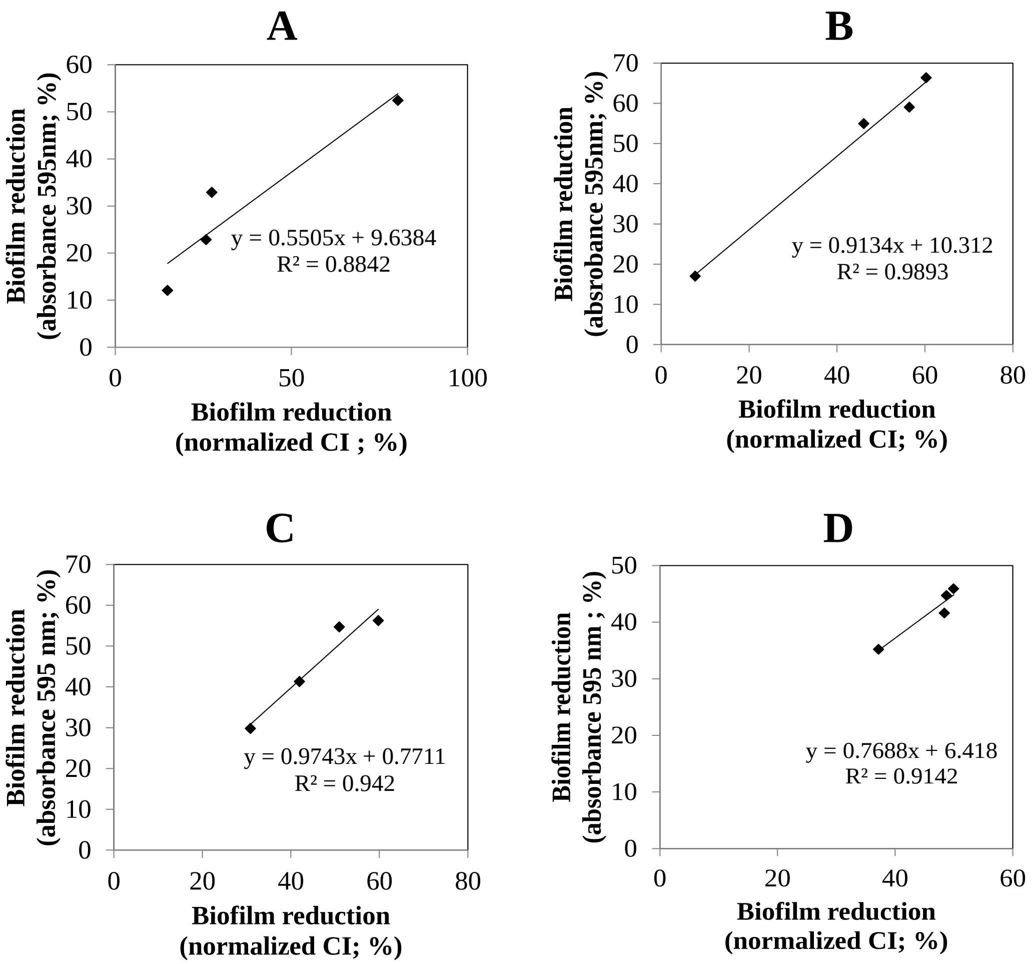

Figure S**3.** Linear regression equations and coefficients of determination (R^2) comparing the percentage of biofilm reduction calculated using normalized CI and absorbance 495 nm values for each biofilm-producing staphylococcal strain: *S. aureus* 15981 (A), *S. aureus* ISP479r (B), *S. aureus* IPLA1 (C), *S. aureus* Sa9 (D).
